# Estimated Annual Economic Burden of Dry Eye Disease Based on a Multi-Center Analysis in China: A Retrospective Study

**DOI:** 10.3389/fmed.2021.771352

**Published:** 2021-12-01

**Authors:** Wanju Yang, Yanzhu Luo, Shangcao Wu, Xiaoxia Niu, Yanshuang Yan, Chen Qiao, Wei Ming, Ying Zhang, Haoyu Wang, Dan Chen, Mengying Qi, Lan Ke, Ying Wang, Liping Li, Shaowei Li, Qingyan Zeng

**Affiliations:** ^1^Aier Eye Hospital of Wuhan University, Wuhan, China; ^2^Aier School of Ophthalmology, Central South University, Changsha, China; ^3^Department of Cornea and Ocular Surface Diseases, Wuhan Aier Hankou Eye Hospital, Wuhan, China; ^4^Department of Cornea and Ocular Surface Diseases, Harbin Aier Eye Hospital, Harbin, China; ^5^Department of Cornea and Ocular Surface Diseases, Guangzhou Aier Eye Hospital, Guangzhou, China; ^6^Aier Cornea Institute, Beijing, China; ^7^Aier School of Ophthalmology and Optometry, Hubei University of Science and Technology, Xianning, China

**Keywords:** dry eye disease, burden of disease, costs, multi-center analysis, China

## Abstract

**Purpose:** To conduct a multi-center analysis and assess the economic burden due to dry eye disease (DED) in China.

**Design:** A retrospective and cross-sectional study.

**Methods:** Patients (*n* = 598) with diagnosed DED were recruited from 3 eye centers (in central, southeast, and northeast China) from 1 January 2018 to 31 December 2018. Data were collected regarding the examination, pharmacological therapy, and non-pharmacological therapy fees. Sub-group analyses were stratified by eye center, DED severity, types of DED, number of visits to physicians, and residential area. A logistic regression analysis was conducted to investigate the variables influencing total costs.

**Results:** The per capita costs devoted to DED at the 3 centers were 422.6, 391.3, and 265.4 USD, respectively. The costs of non-pharmacological therapy accounted the largest part in three centers (75.6, 76.4, 76.5%, respectively). Patients with severe DED sustained the largest economic burden. Patients with mixed type of DED spent the most comparing to patients with either evaporative or aqueous-deficient types of DED. Patients spent more during the first visit compared with subsequent visits. Patients living in urban areas spent significantly more than did those living in rural areas (*P* = 0.001). The logistics regression analysis showed that total costs were significantly influenced by DED severity, number of visits to physicians, and area of residence (beta = 2.83, 0.83, 1.48; *P* < 0.0001).

**Conclusions:** DED is a chronic ocular disease that timely non-cost counseling, early diagnosis, and efficacious treatment can reduce its economic burden on patients and the society.

## Introduction

Dry eye disease (DED) is a chronic and progressive multifactorial disorder of the ocular surface characterized by unstable tear film; or imbalanced microenvironment caused by abnormal tear quality, quantity, and dynamics. It can be accompanied by inflammatory reactions of the ocular surface, tissue damage, and neurological abnormalities, which result in a variety of symptoms of ocular discomfort, visual dysfunction, or both (1). Among people seeking eye care in the United States, DED has become the fifth most prevalent ocular condition in women and ninth most prevalent in men (2).

The prevalence of DED ranges from 5.28 to 33.7% worldwide (3–5). The Dry Eye Workshop (DEWS) committee reported that the prevalence of DED in Southeast Asia is as high as 20.0 to 52.4% (6). In New Zealand, signs of clinical dry eye were present in almost half of a cohort of 45-year-olds (7). In China, a meta-analysis revealed that DED diagnosed by symptoms alone affected 31.40%, or corresponding to 394.13 million affected individuals in the country (4).

DED can be caused by a variety of reasons, including aging, female population (6, 8), environmental factors (e.g., extreme temperature and reduced humidity) (9), and daily-life behaviors [smoking (10), digital screen exposure (11, 12), reduced caffeine consumption, and contact lenses (13)]. Surgical and para-surgical causes such as refractive (14, 15) and cataract surgeries (16, 17) can promote DED, as well as certain medications (18, 19) such as beta-blockers, antihistamines, oral contraceptives, and anti-glaucoma eye drops. A history of thyroid disease, and poorer self-rated health (16), have also been associated with DED.

Many therapies have been demonstrated to improve the signs and symptoms of DED (20). However, global studies suggest that long-term treatment of DED imposes a substantial economic burden on patients and society. In the United States in 2008, the overall burden of DED for the healthcare system was 3.84 billion United States dollars (USD), the cost of managing DED per patient was 11,302 USD, and the overall societal cost was 55.4 billion USD. The costs categories included ocular lubricant treatment, cyclosporine, punctal plugs, physician visits, and nutritional supplements (21). A retrospective study conducted in Germany showed that total costs were ~117 million euro for a DED cohort of 35,026 patients. The costs were analyzed based on the healthcare resource used by the DED patients, including visits to the general practitioner or ophthalmologist, pharmacological treatment, and ocular procedures (uncommon) (22). A systemic literature review evaluated and compared the burden of DED across France, Germany, Italy, Spain, United Kingdom, United States, and Japan (23). Clegg et al. (24) reported that the direct economic burden of managing DED in European countries in 2003 ranged from 270 USD to 1,100 USD per patient and the costs mainly included diagnostic test, specialists visits, prescribed drugs, and surgery. While DED costs vary among countries, the economic burden of DED across regions is comparable. Yet, there is limited data regarding the annual economic burden of DED in Asia, especially China. Besides, seldom research reported the costs of non-pharmacological therapies.

This study aims to conduct a multi-center comprehensive estimation of the costs associated with DED for the year 2018 in China and provides insight into the burden of DED on patients and society.

## Materials and Methods

### Data Source

The study was approved by the ethics committees of 3 eye centers, Wuhan Aier Hankou Eye Hospital (Center-1, central China), Guangzhou Aier Eye Hospital (Center-2, southeast China), and Ha'erbin Eye Hospital (Center-3, northeast China), respectively. The study was conducted in accordance with the tenets of the Declaration of Helsinki, and the enrolled patients provided signed informed consent.

The participants were recruited from outpatients visiting the above 3 eye centers, from 1 January 2018 to 31 December 2018. The inclusion criteria were: (1) subjects aged ≥18 years, (2) had received a diagnosis of DED without other ocular diseases (such as cataract or glaucoma), and (3) were managed and followed-up in the study centers without any interventions from other hospitals or pharmacies. Individuals with any of the following were excluded: (1) eye surgeries in the past 6 months, (2) infectious corneal diseases during follow-up visits, or (3) ended follow-up during the study year.

DED diagnosis and severity criteria were that of the dry eye consensus of the Chinese Medical Association in 2013, and defined as mild, moderate, or severe (Table 1). Classification of DED was consistent with the TFOS (Tear Film and Ocular Surface Society) DEWS II Definition and Classification Report.

**Table 1:** Diagnosis and severity criteria of DED.

**Symptoms**		**Mild subjective symptoms with negative corneal fluorescein staining**
Signs		• Meeting one of the following objective signs: • Tear break-up time ≤5 s, or Schirmer's *I*-test ≤5 mm/5 min; • Tear break-up time >5 s up to 10 s, or Schirmer's *I*-test^*^ between nil up to 5 mm/5 min, with positive corneal and conjunctival fluorescein staining
Severity	Mild	Mild subjective symptoms with negative corneal fluorescein staining
	Moderate	Moderate and severe subjective symptoms with positive corneal fluorescein staining; corneal staining disappeared after treatment
	Severe	Moderate and severe subjective symptoms with positive corneal fluorescein staining; staining did not completely disappear after treatment

* *Schirmer's I-test is without topical anesthesia*.

### Data Collection

The outpatient medical record systems of the 3 hospitals were searched for the cost information of the enrolled patients during the 1-year follow-up. The costs included 3 categories of fees: examinations, pharmacological therapy, and non-pharmacological therapy.

The examination fees included the costs of the general ophthalmological examinations (i.e., intraocular pressure and slit lamp inspection with fluorescein staining) and examinations related to DED (i.e., quantitative and qualitative evaluations of the tear film, and the morphology and function of the meibomian glands). The pharmacological therapy fees consisted of the cost of eye drops to treat DED during the year. Non-pharmacological therapy costs included costs of ophthalmic physiotherapy (i.e., meibomian gland massage, palpebral margin cleaning, eyelid nebulization therapy, and intense pulsed light) and costs of purchased products for treatment (i.e., lacrimal punctual plugs, moisture chamber glasses, corneal bandage lenses, and warm compress eye masks).

### Statistical Analysis

All the data were analyzed using SPSS 22.0 (SPSS IBM, New York, NY, USA) software. A *P* < 0.05 was considered statistically significant. Demographic comparisons among the 3 centers (from the central, southeast, and northeast districts) were performed *via* chi-squared test for dichotomous variables, analysis of variance (ANOVA) for continuous variables, and non-parametric tests for non-normally distributed data.

The subjects were categorized, both overall (the 3 centers) and for each center individually, to determine the respective median values of the total costs.

Comparisons were made of the per capita cost related to DED with the gross domestic product (GDP), and with the income of the city where the medical center is located. Economic burden is shown as USD ($), according to the 2018 currency exchange rate (6.61 Chinese Yuan/USD; National Bureau of Statistics of China, https://data.stats.gov.cn/easyquery.htm?cn=C01&zb=A060J&sj=2018).

In the univariate analyses, cost comparisons between subgroups stratified by different variables were conducted by Mann-Whitney Test (2 categories) or Kruskal-Wallis *H*-Test (multiple categories). The variables included the following: DED severity (mild, moderate, severe); types of DED (evaporative, aqueous-deficient, and mixed DED); number of visits to physicians (first visit, subsequent visit); residential area (urban, rural).

Logistic regression models were used to estimate cost differences between the groups that were lower or higher than the median value. Variables related to DED severity, number of visits to physicians, and residential area were included in the models.

## Results

### Demographics

The study population comprised 598 patients overall, with 199, 199, and 200 patients in Center-1, Center-2, and Center-3, respectively (Table 2). The 3 centers differed significantly by age, gender ratio, and number of visits to physicians (*P* ≤ 0.0001, 0.018, <0.0001). There were 132 mild, 53 moderate, and 14 severe cases of DED in Center-1; 148 mild, 51 moderate, and no severe cases in Center-2; and 142 mild, 40 moderate, and 18 severe cases in Center-3. Regarding to the type of DED, there were 49 evaporative dry eye, 9 aqueous-deficient dry eye, and 141 mixed dry eye subjects in Center-1; 199 mixed dry eye subjects in Center-2; and 37 evaporative dry eye, 96 aqueous-deficient dry eye, and 67 mixed dry eye subjects in Center-3. The number of visits were 1 to 5, 1 to 4, and 4 at Center-1, Center-2, and Center-3.

**Table 2:** Demographic characteristics of patients at 3 eye centers^a^.

	**Center-1**	**Center-2**	**Center-3**	***P***
Subjects	199	199	200	—
Age, y	48.01 ± 13.71	42.22 ± 16.19^*^	47.57 ± 14.11^#^	<0.0001^b^
Gender, female/male	140/59	113^*^/86^*^	130/70	0.018^c^
Numbers of follow-ups (median, range)	2, 1–15	2, 1–31	4, 4–12^*^^#^	<0.0001^d^
Number of visits to physicians (first/subsequent visit)	147/52	137/62	179^*^/21^*^	<0.0001^c^
DED severity (mild/moderate/severe)	132/53/14	148/51/0^*^	142/40/18	<0.0001^c^
Type of DED (evaporative/aqueous-deficient/mixed)	49/9/141	199^*^/0^*^/0^*^	37/96^*^^#^/67^*^^#^	<0.0001^c^
Residential area (rural/urban)	64/135	37^*^/162^*^	68/132	0.001^c^

a *Data is reported as n, unless indicated otherwise*;

b *ANOVA*;

c *chi-squared test*;

d *Kruskal-Wallis H*.

* *Compare with Center-1, P < 0.05*;

# *Compare with Center-2, P < 0.05; Bonferroni test was performed*.

*Center-1, Wuhan Aier Hankou Eye Hospital, Wuhan, central China; Center-2, Guangzhou Aier Eye Hospital, Guangzhou, southeast China; Center-3, Ha'erbin Aier Eye Hospital, Ha'erbin, northeast China*.

### Per Capita Annual Costs Analysis

The per capita total costs related to DED at Center-1, Center-2, and Center-3 were $422.6, $391.3, and $265.4 (*P* = 0.043), respectively (Table 3). The percentages in per capita residual income were 3.16, 2.31, 2.44%; and GDP (year 2018) were 1.66, 1.05, and 2.65%.

**Table 3:** Percentages of annual per capita DED costs in residual income and GDP (year 2018)^a^.

**Annual per capita DED costs**	**Center-1**	**Center-2**	**Center-3**	***P*** ^b^
Examination fee	$53.0 ± 35.5 (12.5%)	$36.9 ± 30.1 (9.4%)^*^	$29.3 ± 24.5 (11.0%)^*^^#^	**<0.0001**
Pharmacological therapy fee	$50.1 ± 51.0 (11.9%)	$55.5± 55.1 (14.2%)^*^	$33.1 ± 21.6 (12.5%)^#^	**<0.0001**
Non-pharmacological therapy fee	$319.5 ± 302.6 (75.6%)	$298.9 ± 382.9 (76.4%)	$203.1 ± 129.5 (76.5%)	0.098
Total fee	$422.6 ± 324.8	$391.3 ± 422.6	$265.4 ± 145.0^*^	**0.014**
Per capita residual income in each area^c^	$13,362.6 (3.2%)	$16,919.7 (2.3%)	$10,857.9 (2.4%)	–
Per capita GDP^d^	$25,417.1 (1.7%)	$37,278.6 (1.1%)	$10,017.2 (2.7%)	–

* *Compare with Center-1, P < 0.05*;

# *Compare with Center-2, P < 0.05; Bonferroni test was performed*.

a *Reported as US dollars ($), the 2018 currency exchange rate 6.61 Chinese Yuan/USD*;

b *Kruskal-Wallis H-test*;

c *total fees related to residual income in each area*;

d *total fees related to per capita GDP. The bold values means there was significant difference*.

The examination fees differed significantly among the centers (*P* < 0.0001), as well as the pharmacological therapy fees (*P* < 0.0001), but the non-pharmacological fees were comparable (*P* = 0.098). The costs of non-pharmacological therapy accounted the largest part in three centers (75.6, 76.4, 76.5, respectively).

### Sub-Group Analysis

The annual (2018) per capita costs related to DED among the subgroups was reported (Table 4). Compared with patients with mild or moderate DED, those with severe DED incurred the highest examination, pharmacological therapy, and non-pharmacological therapy fees. Compared with patients with evaporative or aqueous-deficient DED, those with mixed DED incurred the highest examination, non-pharmacological therapy, and total fees. There was no significant difference on the pharmacological therapy fee among three types of DED. Patients spent significantly more at the first visit on the examination fee, pharmacological therapy fee, non-pharmacological therapy fee, and the total fee (*P* =0.010, 0.049, <0.0001, <0.0001). Patients living in urban areas spent more than those living in rural areas on the examination fee, pharmacological therapy fee, non-pharmacological therapy fee, and the total fee (*P* <0.0001, <0.0001, <0.0001, <0.0001).

**Table 4:** Summary of the annual (2018) per capita costs related to DED among the subgroups (USD).

	**Examination fee**	**Pharmacological therapy fee**	**Non-pharmacological therapy fee**	**Total fee**
**DED severity**				
Mild	$35.6 ± 31.3 (13.8%)	$36.9 ± 32.6 (14.3%)	$186.3 ± 191.8 (72.0%)	$258.4 ± 214.7
Moderate	$49.0 ± 31.0 (8.3%)^*^	$67.2 ± 65.6 (11.3%)^*^	$476.7 ± 398.7 (80.4%)^*^	$592.2 ± 429.6^*^
Severe	$51.9 ± 32.5 (8.1%)^*^	$74.2 ± 49.8 (11.6%)^*^	$513.1 ± 300.8 (80.3%)^*^	$639.1 ± 291.6^*^
*P* ^a^	<0.0001	<0.0001	<0.0001	<0.0001
**Types of DED**				
Evaporative	$35.9 ± 31.2 (9.7%)	$49.3 ± 54.0 (13.3%)	$286.4 ± 354.1 (77.1%)	$371.6 ± 391.7
Aqueous-Deficient	$32.1 ± 25.8 (14.9%)	$41.5± 39.9 (19.3%)	$141.6 ± 77.8 (65.8%)^∧^	$215.2 ± 116.9
Mixed	$48.7 ± 33.6 (11.7%)^∧^^#^	$44.4 ± 35.8 (10.7%)	$323.0 ± 254.5 (77.6%)^∧^^#^	$416.1 ± 272.1^∧^^#^
*P* ^a^	<0.0001	0.800	<0.0001	<0.0001
**Number of visits to physicians**				
First visit	$40.8 ± 30.1 (10.7%)	$47.6 ± 46.4 (12.4%)	$294.3 ± 305.0 (76.9%)	$382.7 ± 338.9
Subsequent visit	$36.0 ± 37.3 (12.8%)	$41.4 ± 44.5 (14.8%)	$203.1 ± 247.3 (72.4%)	$280.6 ±259.9
*P* ^b^	0.010	0.049	<0.0001	<0.0001
**Residential area**				
Rural area	$33.5 ± 30.9 (12.7%)	$38.3 ± 41.7 (14.5%)	$192.5 ± 201.7 (72.8%)	$264.3 ± 227.8
Urban area	$42.2 ± 32.0 (10.6%)	$49.3 ± 47.3 (12.4%)	$305.7 ± 319.3 (77.0%)	$397.2 ± 349.7
*P* ^b^	<0.0001	<0.0001	<0.0001	<0.0001

a *Kruskal-Wallis H-test*;

b *Mann-Whitney Test*;

* *Compare with Mild, P < 0.05 and Bonferroni was performed; ∧Compare with Evaporative, P < 0.05*,

# *Compare with Aqueous-deficient, P < 0.05, and Bonferroni test was performed*.

### Logistics Regression Analysis

The logistics regression analysis showed that, for the centers overall, DED severity, number of visits to physicians, and residential area had a significant influence on total costs (beta = 2.83, 0.83, 1.48, respectively, *P* < 0.0001). The significant variable that influenced total costs at Center-1 was disease severity only (beta = 1.37, *P* < 0.0001); at Center-2, the influential variables were disease severity (beta = 2.84, *P* < 0.0001) and number of visits to physicians (beta = 1.51, *P* = 0.003); at Center-3, disease severity (beta = 2.84, *P* < 0.0001) and residential area (beta = 1.65, *P* = 0.004) were of greatest importance.

## Discussion

To the best of our knowledge, this is the first multi-center analysis of the annual economic burden imposed by DED in China. The global economic burden due to DED, to the patient and society, is not trivial. In the last decade, various methods have been used to analyze the economic burden on patients with DED worldwide (Table 5) (21, 22, 25, 27–30), but little is known about the situation in China (31).

**Table 5:** Studies on the economic burden of DED published between 2010 and 2020.

	**Year, location**	**Methods**	**Study population**	**Cost analysis**
**North America**				
Yu et al. (21)	2008, USA	Survey, cross-sectional study, decision analytic model	2171 DED Pts	• Direct PCC: $783 • Indirect PCC: $11,302
Galor et al. (25)	2001–2006, USA	Survey, retrospective study	147 DED Pts	• DED medication PCC: • 2001–2002, $55/y • 2003–2004, $137/y • 2005–2006, $299/y • Yearly overall, $217.3 ± 23.4
**Europe**				
Tachkov et al. (26)	2016–2017, Sofia	Prospective, observational, decision tree analysis	64 eyes with POAG & DED	Median costs of DED treatment €179.9 ± 9.4
Darbà et al. (27)	1997–2015, Spain	Multi-centers, insurance claims analysis, retrospective study	36,081 DED Pts	• Total costs: increased from €4.9 to €30.3 M during the study period • Mean annual cost per Pt: €7,379 increase
Siffel et al. (22)	2008–2015, Germany	Insurance claims analysis, retrospective study	85,560 DED Pts with at least one confirmed diagnosis	• Total PCC: €3,809 ± 8,195 • Outpatient visits PCC: €932 ± 1,229 • Acute day ward visits PCC: €50 ± 328 • Pharmacological treatments PCC: €816 ± 3,415
**Asia**				
Mizuno et al. (28)	2005–2008, Japan	Multi-center, survey, cross-sectional study	118 DED Pts	• Annual PCC: $530 • Clinical PCC: $165 • Pharmacological PCC: $323 • Punctual plug PCC: $42
Nagata et al. (29)	2014–2015, Japan	Survey, cross-sectional study	266 subjects with eye condition including DED	Annual PCC of eye conditions including DED: $253.3
Waduthantri et al. (30)	2008–2009, Singapore	Pharmacy & clinic inventory database analysis, retrospective	54,052 DED Pts	• 2008, $22.11 • 2009, $23.59
Yao and Le (31)	2016, SE China	Survey, cross-sectional study	34 SSDE & 30 non-SSDE subjects	• Annual Medication costs: SSDE ¥7,637.2 ± 6,079.0, non-SSDE ¥1,179.1 ± 990.4 • Annual out-of-pocket money: SSDE ¥2,627.8 ± 1,857.0, non-SSDE ¥481.9 ± 393.3 • Annual indirect expense: SSDE ¥828.0 ± 1,866.0, non-SSDE ¥487.2 ± 1,404.0 • Expense on auxiliary therapy: SSDE ¥2,757.1 ± 2,496.0
Present study, 2021	2018, C, SE, NE China	Multi-centers, outpatient medical record system analysis, retrospective study	598 DED Pts	• Total PCC: $422.6, $391.3, and $265.4 in 3 centers • Sub-groups: • Severity: mild cf. moderate cf. severe = mild $258.4 ± 214.7; moderate $592.2 ± 429.6; severe $639.1 ± 291.6 • Types: evaporative cf. aqueous-deficient cf. mixed= evaporative $371.6 ± 391.7; moderate $215.2 ± 116.9; severe $4,616.1 ± 272.1 • Visit: first $382.7 ± 338.9; subsequent $280.6 ± 259.9 • Residence: rural $264.3 ± 227.8; urban $397.2 ± 349.7

*C, central; NE, northeast; PCC, per capita cost; Pt, patient; SE, southeast*.

In the current study, data were collected from 3 eye centers, in central, southeast, and northeast China, respectively. The annual total costs (per capita) associated with DED ranged from $264.5 to $422.6, which appeared lower than the studies discussed above. However, the economic and medical status in different regions is an important factor that affects economic burden. We found that the per capita costs of DED accounted for 2.31–3.16% of per capita residual income, and 1.05–2.65% of per capita GDP, in the different districts. When taking into account the prevalence of DED in China (affecting 394.13 million individuals) (4), the overall burden of DED for the healthcare system translates to $104.2 billion to $166.6 billion per year, which was much higher than the estimated annual economic burden in the US society overall [$55.4 billion (21)]. Therefore, the economic burden of DED on the Chinese people is such that more reasonable policymaking is needed regarding state-set medication prices and medical care reimbursement.

There were significant differences in the annual per capital DED costs, examination fee, and pharmacological therapy fee among three centers. The non-pharmacological therapy fee was still higher in Center-1 than other two centers, although the difference was not significant. One of the reasons is that the DED specialized outpatient clinic was set in Center-1 in 2014 and patients in Wuhan city were more willing to accept complicated examinations and non-pharmacological therapies. Costs in Center-3 located in Ha'erbin were all much lower than Center-1 and Center-2 as the city was less developed and the overall outpatient costs were much lower.

As far as we know, this study is the first multi-center analysis to report non-pharmacological costs, that is, those associated with ophthalmic physiotherapy conducted in clinics and those treatment products purchased. The non-pharmacological costs accounted for the largest share of the total costs in all three centers to ($319.5, $298.9, $203.1, 75.6, 76.4, 76.5%, respectively). Examples of ophthalmic physiotherapy conducted in clinics are meibomian gland expression (MGX), palpebral margin cleaning, eyelid nebulization therapy, and intense pulsed light (IPL) treatment. Treatment products purchased by patients include lacrimal punctual plugs, moisture chamber glasses, corneal bandage lens, and warm compress eye masks. Among them, MGX and IPL were most widely used according to our preliminary single center analysis (32). As meibomian gland disease related DED becomes more common, technologies for efficacious management are important (33) and IPL was found safe for DED therapy (34). However, they are currently quite costly, and more advances in methods and strategies for DED management that may reduce costs are warranted.

From the logistic regression analysis of the current study, it was found that the total costs of the patient were significantly influenced by DED severity. Compared with patients with mild or moderate DED, those with severe DED incurred the highest medical expense. Although DED is not curable, timely and efficient treatment can relieve the symptoms. However, one study found that patients tended to discontinue follow-ups as DED prolonged after diagnosis (35). Thus, timely and comprehensive education on patient is essential to prevent their discouragement on following treatment. Besides, early intervention is important to prevent DED progression, which can potentially improve quality of life and work productivity, reducing the indirect costs of DED in more severe stages.

Patients with mixed type of DED spent the most and then followed patients with evaporative and aqueous-deficient DED. The possible reason was that the symptoms of patients with aqueous-deficient DED could be alleviated by pharmacological therapies which were much inexpensive. While patients with mixed or evaporative DED were more likely to be treated with non-pharmacological therapies or combining multiple kinds of therapied which could result in more costs.

In addition to DED severity and type, we also found that number of visits to physicians influenced the costs. Although much less subjects had follow-up visits, the costs of subsequent visits were comparable to the costs of subjects' first visit, especially the examinations and pharmacological therapy fee. As DED is not curable and in need of long-term treatment to alleviate symptoms, further research is essential to investigate on cheaper examination technologies and more efficient therapies so that patients could spend less on follow-up visits during their lifetime. Besides, in China, some costs on DED non-pharmacological therapy were not covered by patients' insurance. Thus, the support from the government and insurance companies is also important to reduce the burden on patients.

Compared with patients living in rural areas, those living in urban areas spent more on DED examinations and treatment. It is reasonable that patients with these later characteristics may be more annoyed by DED and more willing to seek medical treatment. In addition, such patients are more likely to experience longer exposures to electronic devices, which is a risk factor of DED onset and deterioration. Therefore, for these subjects, more detailed non-cost counseling is necessary to prevent disease progression.

Our study has several strengths. It gains credibility by being multicenter, with 3 eye centers in central, southeast, and northeast China. This aspect makes it more representative and comprehensive. Another strength is that the cost data was collected not from self-questionnaires, but from the outpatient medical record systems of the hospitals, which is more objective. Finally, subgroup and logistic regression analyses were conducted to investigate the significance of the variables that influence the costs associated with DED. However, there are also some limitations. One limitation is that only the direct medical costs related to DED were collected or calculated. The effect of indirect costs, such as low employment, absence from work, and impaired productivity, will be investigated in the future. Another factor of note is that the actual economic burden of DED could have been underestimated, as we only collected data for 1 year. Future study using decision tree or Markov model is needed. Besides, the sample size was relatively small and we only included centers in China which may influence the generalizability of our study outside China.

## Conclusions

Patients due to DED sustain a heavy economic burden for the healthcare system in China, which translates to $104.2–$166.6 billion per year when taking into account the prevalence of DED in China (affecting 394.13 million individuals). DED is a chronic ocular disease that costs more on patients in more severe disease stage. Thus, timely non-cost counseling, early diagnosis, and efficacious treatment are essential to retard the disease progression and potentially reduce the economic burden.

## Data Availability Statement

The raw data supporting the conclusions of this article will be made available by the authors, without undue reservation.

## Ethics Statement

The studies involving human participants were reviewed and approved by Ethics Committees of Wuhan Aier Hankou Eye Hospital (Center-1), Guangzhou Aier Eye Hospital (Center-2), and Ha'erbin Eye Hospital (Center-3). The patients/participants provided their written informed consent to participate in this study.

## Author Contributions

WY, YL, SL, and QZ conceived and designed research. YL, XN, YY, CQ, WM, YZ, HW, DC, MQ, LK, YW, and LL collected data and conducted research. WY and SW analyzed and interpreted data. WY and QZ wrote the initial paper. SL and QZ revised the paper. QZ had primary responsibility for final content. All authors read and approved the final manuscript.

## Funding

This work was supported by the Aier Eye Hospital Group (Grant No. AR1904D2), Youth Project of the Medical Scientific Research Foundation of Wuhan Municipal Health Commission (Grant No. WX21Q33), the Science and Technology Innovation Program of Hunan Province (Grant No. 2020SK50104), and Scientific Research Fund project of Aier Eye Hospital Group (Grant No. AF2004D10). The authors declare that the funding body was not involved in study design, data collection, analysis, interpretation, and writing of the study.

## Conflict of Interest

The authors declare that the research was conducted in the absence of any commercial or financial relationships that could be construed as a potential conflict of interest.

## Publisher's Note

All claims expressed in this article are solely those of the authors and do not necessarily represent those of their affiliated organizations, or those of the publisher, the editors and the reviewers. Any product that may be evaluated in this article, or claim that may be made by its manufacturer, is not guaranteed or endorsed by the publisher.

## Abbreviations

ANOVA: analysis of variance
DED: dry eye disease
DEWS: The Dry Eye Workshop
GDP: gross domestic product
SSDE: Sjögren's syndrome dry eye
TFOS: Tear Film and Ocular Surface Society
USD: United States dollars.
